# Effect of transverse thoracic muscle plane block on postoperative cognitive dysfunction after open cardiac surgery: A randomized clinical trial

**DOI:** 10.1111/jcmm.17710

**Published:** 2023-03-06

**Authors:** Shibiao Chen, Hua Zhang, Yang Zhang

**Affiliations:** ^1^ Department of Anesthesiology First Affiliated Hospital of Nanchang University Nanchang China; ^2^ Department of Anesthesiology Nanchang Hongdu Hospital of TCM Nanchang China

**Keywords:** open cardiac surgery, postoperative cognitive dysfunction, insulin resistance, the length of hospital stay, transversus thoracis muscle plane

## Abstract

The transversus thoracis muscle plane (TTMP) block provides effective analgesia in cardiac surgery patients. The aim of this study was to assess whether bilateral TTMP blocks can reduce the incidence of postoperative cognitive dysfunction (POCD) in patients undergoing cardiac valve replacement. A group of 103 patients were randomly divided into the TTM group (*n* = 52) and the PLA (placebo) group (*n* = 51). The primary endpoint was the incidence of POCD at 1 week after surgery. Secondary outcome measures included a reduction of intraoperative mean arterial pressure (MAP) >20% from baseline, intraoperative and postoperative sufentanil consumption, length of stay in the ICU, incidence of postoperative nausea and vomiting (PONV), time to first faeces, postoperative pain at 24 h after surgery, time to extubation and the length of hospital stay. Interleukin (IL)‐6, TNF‐α, S‐100β, insulin, glucose and insulin resistance were measured at before induction of anaesthesia, 1, 3and 7 days after surgery. The MoCA scores were significantly lower and the incidence of POCD decreased significantly in TTM group compared with PLA group at 7 days after surgery. Perioperative sufentanil consumption, the incidence of PONV and intraoperative MAP reduction >20% from baseline, length of stay in the ICU, postoperative pain at 24 h after surgery, time to extubation and the length of hospital stay were significantly decreased in the TTM group. Postoperatively, IL‐6, TNF‐α, S‐100β, HOMA‐IR, insulin, glucose levels increased and the TTM group had a lower degree than the PLA group at 1, 3 and 7 days after surgery. In summary, bilateral TTMP blocks could improve postoperative cognitive function in patients undergoing cardiac valve replacement.

## 1 INTRODUCTION

Postoperative cognitive dysfunction (POCD) is considered as a common neurologic complication in elderly patients undergoing open cardiac surgery.^1^ The incidence of POCD widely varies between 30% and 60% 1 week after open cardiac surgery and it has been consistent over the years compared with other complications following cardiac surgery.^2^, ^3^ The cognitive dysfunction incidence has become an intriguing issue. The occurrence of POCD causes several adverse effects, such as increased postoperative morbidity and mortality, prolonged hospitalization, reduced quality of life of patients and causes a serious burden on individuals and the healthcare system.

The increasing number of elderly patients undergoing surgery has led to an increased incidence of POCD.^4^ However, the pathogenesis of POCD is still not fully understood and patients still lack effective treatment.

Over the last decade, various methods are used to prevent and treat POCD in cardiac patients with the increasing understanding of the mechanisms involved. A large number of studies have shown that application of high‐dose opioids, hyperglycemia, inflammation, cardiopulmonary bypass (CPB), the formation of cerebral microemboli, anaesthesia and hypoxia are some mechanisms suggested for POCD.^5^, ^6^ Glumac et al.^7^ showed that preoperative administration of dexamethasone reduced the incidence and severity of inflammatory response prompted by cardiac surgery, and thus ameliorate cognitive decline following cardiac surgery. Our previous research^8^, ^9^ have demonstrated that bilateral transversus thoracis muscle plane (TTMP) blocks can provide good perioperative analgesia and promote postoperative recovery in patients undergoing open cardiac surgery by reducing postoperative insulin resistance, systemic inflammation and perioperative sufentanil consumption. However, studies concerning the effects of TTMP block on cognitive function in patients undergoing open cardiac surgery are not available.

Therefore, we hypothesized that the incidence of POCD at 1 week after surgery would be lower in patients who received TTMP blocks compared with those who received placebo. The aim of this study was to assess whether bilateral TTMP blocks can reduce the incidence of POCD in patients undergoing cardiac valve replacement under CPB.

## 2 METHODS

This randomized, controlled study was conducted at First Affiliated Hospital of Nanchang university. After approval by the hospital's Clinical Research Ethics Committee and written informed consent was obtained from all patients participating in our study. Then it was registered in the Chinese Clinical Trial Registry (registration number ChiCTR 2200058987). Our study was completed from 1 March 2022 to 26 July 2022.

The inclusion criteria of this study included people aged over 65 years old, American Society of Anesthesiologists physical status II/III, body mass index of 18–25 kg/m^2^ and preoperative Mini‐Mental State Examination (MMSE) score ≥23. Criteria for exclusion in our trial was as follows: mental disorder, acute or chronic infectious diseases, an allergy to local anaesthetics, secondary surgery, severe deafness or vision problems, liver dysfunction, illiteracy, a diagnosis of diabetes mellitus, urgent surgery, hemodynamic instability and communication difficulties related to pronunciation or dialect.

### 2.1 Surgery and anaesthesia

After entering the operating room, electrocardiography, oxyhemoglobin saturation, the mean arterial pressure and central temperature were monitored. Anaesthesia was induced with sufentanil, midazolam, etomidate and rocuronium and subsequently maintained with sufentanil, propofol and rocuronium according to the clinical needs in all patients. The depth of anaesthesia was monitored by a bispectral index and it was maintained between 45 and 55 intraoperatively. All patients underwent biological valve replacement with CPB via a median sternotomy. Patient‐controlled intravenous analgesia with sufentanil and flurbiprofen axetil was used to perform postoperative analgesia and 50 mg flurbiprofen axetil was injected i.v. at 6 h intervals as a supplementary analgesic. We selected the surgeries of the same group of surgeons in our study. All patients were sent to the ICU after the surgery.

### 2.2 Randomization and blinding

All enrolled patients were randomized (1:1) to receive TTMP block (TTM group) or placebo (PLA group) using a computer‐generated random number and was kept in the sealed envelopes by the attending anaesthesiologist. Another researcher prepared the trial medication (the normal saline or 0.30% ropivacaine) according to the group allocation of the sealed envelopes. The operator who performed bilateral TTMP blocks did not know whether the fluid is normal saline or ropivacaine. The treating physicians, patients, nurses, anaesthesiologists and the investigators were blinded for treatment allocation. This was a double‐blind, randomized, controlled study.

### 2.3 Ultrasound‐guided TTMP block

The TTMP blocks were performed in the operating room using high‐frequency linear ultrasound probe (Wisonic). The probe was placed lateral to the sternal border at the anterior T4‐T5 interspace, then we could find the pectoralis major muscle, the external intercostal muscle and the transversus thoracis muscle.^10^ A nerve block needle was located in the fascial plane between the intercostal muscle and the transversus thoracic muscle and 0.30% ropivacaine (20 mL) was injected into this plane. All nerve blocks were completed by the same skilled anaesthesiologist within 20 min.

### 2.4 Clinical and biochemical parameters

The primary outcome measures of our study was the incidence of POCD at 1 week after surgery. The Beijing version of the Montreal Cognitive Assessment (MoCA‐BJ) was analysed for assessing the cognitive function ranged from 0 to 30 scores.

Secondary outcome measures included a reduction of intraoperative mean arterial pressure (MAP) >20% from baseline, intraoperative and postoperative sufentanil consumption, length of stay in the ICU, incidence of postoperative nausea and vomiting (PONV), time to first faeces, postoperative pain at 24 h after surgery, time to extubation and the length of hospital stay. Interleukin (IL)‐6, TNF‐α, S‐100β, insulin, glucose and insulin resistance were measured at before induction of anaesthesia, 1, 3, 7 days after surgery. The blood sample was immediately centrifuged to separate the serum. Then it was frozen at −70°C for subsequent analysis. Insulin resistance was assessed by the homoeostasis model assessment, that is, HOMA‐IR = blood glucose (mmol/L) × blood insulin (munits/mL)/22.5. Postoperative pain was measured using the Numerical Rating Scale (NRS) score (range from 0 to 10). The MoCA‐BJ scores were evaluated at 1 day before operation as well as 1 week after operation.

### 2.5 Statistical analysis

The sample size was calculated according to comparison of the primary endpoint of the MoCA‐BJ scores at 1 week after surgery on the basis of a pilot study (*n* = 14 patients in per group). An estimated sample size of 48 patients in each group were needed with a type I error of *α* = 0.05, a type II error of *β* = 0.1 and a power of 90%. We finally included 20% more patients for analysis to compensate for possible dropout in our trial (*n* = 57 in each group).

The Kolmogorov–Smirnov test was used to verify whether the data conforms to the normal distribution. Continuous data were expressed as the mean ± standard deviation. The Chi‐square or Fisher's exact test were used to analyse categorical data. Biochemical data were evaluated by anova for repeated measurements. The qualitative data were expressed as the frequency and percentage. Student's *t* test was used to assess the intergroup differences with normal distribution, whereas the Wilcoxon–Mann–Whitney test was used to assess the differences in the non‐normally distributed data. A probability value of less than 5% was considered significant.

Statistical analysis was performed using SPSS software (version 17.0).

## 3 RESULTS

**FIGURE 1:**
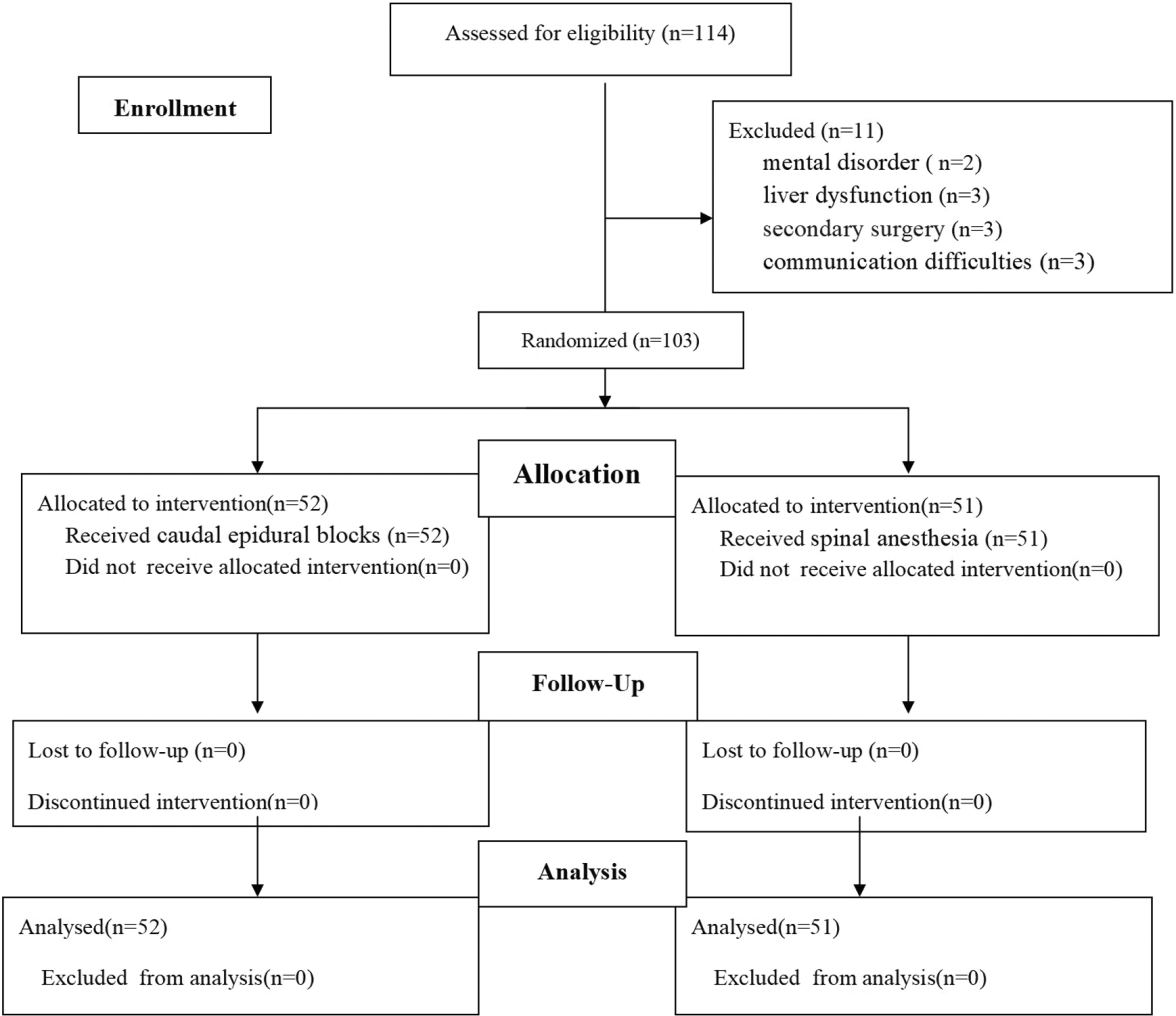
Patient flow diagram.

A total of 114 patients completed the trial. Five patients from the TTM group and six patients from the PLA group were excluded from the study for the following reasons: mental disorder (two), liver dysfunction (three), secondary surgery (three) and communication difficulties (three). Finally, date for 103 patients were finally analysed with 52 in the TTM group and 51 in the PLA group (Figure 1). Baseline characteristics showed no statistically significant differences between TTM group and PLA group (Table 1).

**TABLE 1:** Demographic, clinical and surgical characteristics.

	TTM group (*n* = 52)	PLA group (*n* = 51)	*p*‐Value
Age (years)	72.3 ± 7.1	71.5 ± 6.3	0.69
Body mass index (kg/m^2^)	21.9 ± 3.4	21.5 ± 3.9	0.75
ASA classification (III/IV)	30/22	28/23	0.47
Duration of surgery (min)	187.8 ± 42.3	190.6 ± 36.7	0.55
Cardiopulmonary bypass time (min)	67.5 ± 21.8	70.2 ± 20.3	0.67
Intraoperative bleeding volume (mL)	756.8 ± 217.5	722.9 ± 225.7	0.49
Intraoperative urine output (mL)	879.6 ± 219.3	903.7 ± 215.9	0.62
Sex (male/female)	21/31	25/26	0.73
Procedure			
Mitral valve replacement	28	23	0.42
Aortic valve replacement	24	28	

Compared with the day before surgery, the MoCA scores were significantly decreased at 1 week after surgery in all patients (*p* < 0.05) (Table 2). The MoCA scores were significantly lower and the incidence of POCD decreased significantly in TTM group compared with PLA group at 7 days after surgery (*p* < 0.05) (Table 2). Intraoperative and postoperative sufentanil consumption, the incidence of PONV, the incidence of intraoperative MAP reduction >20% from baseline were significantly lower in the TTM group than in the PLA group (Table 3). Length of stay in the ICU, postoperative pain at 24 h after surgery, time to extubation and the length of hospital stay were significantly decreased in the TTM group compared with the PLA group (Table 3).

**TABLE 2:** Comparison of POCD incidence and MoCA scores in two groups.

	One day preoperation	One week postoperation	Case of POCD	Incidence (%)
TTM group (*n* = 52)	28.62 ± 1.41	26.49 ± 1.87^a^ ^,^ ^b^	8	15.4^c^
PLA group (*n* = 51)	28.65 ± 1.36	23.42 ± 1.75^a^ ^,^ ^b^	16	31.4^c^

^a^ *p* < 0.05 versus PLA Group.

^b^ *p* < 0.05 versus 1 day preoperation.

^c^ *p* < 0.05 versus PLA Group.

**TABLE 3:** Intra‐ and post‐operative clinical outcomes.

	PLA group (*n* = 51)	TTM group (*n* = 52)	*p*‐Value
Intraoperative sufentanil consumption (μg)	109 ± 27	73 ± 12	<0.01
Postoperative sufentanil consumption (μg)	113 ± 26	65 ± 10	<0.01
Reduction >20% from baseline of MAP	22 (42.3%)	10 (19.6%)	<0.05
Postoperative pain at 24 h after surgery	3.7 ± 1.2	2.1 ± 0.9	<0.05
Time to extubation (h)	10.3 ± 4.2	3.4 ± 2.1	<0.01
Time to drain removal (h)	42.9 ± 12.5	40.8 ± 15.4	0.54
Length of stay in the ICU (h)	28.6 ± 9.4	15.6 ± 5.7	<0.05
Incidence of PONV (%)	7 (13.1%)	15 (28.8%)	<0.05
Time to first faeces (h)	39.7 ± 15.6	38.6 ± 13.7	0.65
Length of hospital stay (h)	231.5 ± 21.8	181.3 ± 19.8	<0.05

*Note*: *p* < 0.05 considered statistically significant.

There were no significant differences between the groups in time to first faeces and time to drain removal (Table 3).

There were no significant differences in the serum levels of S‐100β, TNF‐α, IL‐6, insulin, glucose, HOMA‐IR between both groups at base value (Table 4). Postoperatively, IL‐6, TNF‐α, S‐100β, HOMA‐IR, insulin, glucose levels increased and the TTM group had a lower degree than the PLA group at 1, 3 and 7 days after surgery (Table 4).

**TABLE 4:** Measures of blood markers and insulin resistance.

	Baseline	One day after surgery	Three days after surgery	Seven days after surgery
Insulin (units/L)				
PLA group	10.97 ± 1.38	23.43 ± 4.73*	20.53 ± 3.69*	18.35 ± 2.49*
TTM group	11.02 ± 1.25	16.32 ± 3.57*	15.17 ± 2.21*	15.23 ± 1.26*
Glucose (mmol/L)				
PLA group	5.07 ± 2.39	7.45 ± 3.13*	6.48 ± 2.97*	6.35 ± 1.85*
TTM group	5.17 ± 2.23	5.74 ± 2.39*	5.48 ± 1.99*	5.27 ± 1.65*
HOMA‐IR				
PLA group	2.47 ± 0.63	7.75 ± 0.75*	5.91 ± 0.57*	5.17 ± 0.71*
TTM group	2.53 ± 0.54	4.14 ± 0.64*	3.69 ± 0.48*	3.56 ± 0.47*
IL‐6 (pg/mL)				
PLA group	24.57 ± 6.34	105.79 ± 12.55*	97.43 ± 9.54*	79.49 ± 10.47*
TTM group	23.43 ± 7.25	75.37 ± 11.53*	65.92 ± 7.49*	49.87 ± 6.74*
TNF‐α				
PLA group	22.54 ± 3.25	76.74 ± 8.24*	59.67 ± 7.25*	42.73 ± 5.37*
TTM group	22.37 ± 3.17	56.83 ± 7.84*	43.76 ± 6.35*	31.66 ± 4.52*
S‐100β				
PLA group	1.39 ± 0.37	2.18 ± 0.39*	2.02 ± 0.27*	1.89 ± 0.35*
TTM group	1.41 ± 0.35	1.75 ± 0.27*	1.63 ± 0.26*	1.52 ± 0.28*

* *p* < 0.05 considered statistically significant.

## 4 DISCUSSION

This randomized study in 103 cardiac surgery patients demonstrated that the use of TTMP block could reduce perioperative sufentanil consumption, the incidence of PONV, the incidence of intraoperative MAP reduction >20% from baseline, length of stay in the ICU, postoperative pain scores at 24 h after surgery, time to extubation, systemic inflammation and postoperative insulin resistance. Furthermore, these results might be the basis for reducing incidence of POCD, the MoCA scores after surgery and length of hospital stay.

The most commonly used neuropsychological tests for POCD are MMSE and MoCA. Many researchers used MMSE to measure cognitive dysfunction in previous studies.^11^ However, MMSE was difficult to detect the slight cognitive dysfunction with the simplified questions as the ‘ceiling effect’.^12^ Therefore, MMSE was only used for preliminary screening in our study. Our trial used the MoCA to balance the influences of education levels and various ages^13^ and the sensitivity and specificity of the MoCA are superior to MMSE.^14^, ^15^

Barth et al.^16^ found that the great trauma of sawing the sternum and CPB lead to severe postoperative insulin resistance and systemic inflammation. Many studies suggested that insulin resistance is associated with the increase of inflammatory mediator release and the incidence of POCD.^17^, ^18^ Our study demonstrated that ultrasound‐guided TTMP block can reduce the degree of hyperglycaemia and insulin resistance in patients undergoing open cardiac surgery. It was also found that the incidence of POCD had a downward trend and the levels of inflammatory factors S100β, IL‐6 and TNF‐α were significantly decreased compared with the PLA group. Glumac et al.^7^ also found that dexamethasone can reduce the incidence and severity of inflammatory response in patients undergoing cardiac surgery, and thus ameliorate cognitive decline following cardiac surgery. The reduction of insulin resistance was associated with a decreased inflammatory mediator release in our study. Reduced inflammatory response and insulin resistance in the TTM group might be the basis for the decreased incidence of POCD.

Previous studies demonstrated that education years, surgical trauma, acute postoperative pain, age and anaesthesia process were risk factors of cognitive dysfunction.^19^ It has also been reported that the application of opioids may influence the cognitive function of older patients.^20^ Our study found that ultrasound‐guided TTMP block can reduce perioperative sufentanil consumption, the incidence of intraoperative MAP reduction >20% from baseline and postoperative pain scores at 24 h after surgery. These results suggest that TTMP block can reduce the consumption of opioids, maintain intraoperative hemodynamic stability and provide effective analgesia in cardiac surgery patients, which are important in improving cognitive dysfunction. The current study found that TTMP block can reduce the incidence of POCD and have higher MoCA scores 1 week after surgery.

This study has several limitations. First, the patients in our study were followed up to 1 week after surgery. In further study, a longer follow‐up period should be evaluated. Second, we only used two common cognitive tests in our study, and we need to perform other tests in further studies. Finally, this study was conducted in a single centre with small sample size. Therefore, further study required large sample sizes.

## 5 CONCLUSIONS

Our study found that the use of ultrasound‐guided TTMP block could improve postoperative cognitive function in cardiac surgery patients, which may be associated with reduced insulin resistance, systemic inflammation, the perioperative sufentanil consumption, maintain intraoperative hemodynamic stability and provide effective analgesia.

## AUTHOR CONTRIBUTIONS

**Yang Zhang:** Conceptualization (equal); data curation (equal); formal analysis (equal); funding acquisition (equal); investigation (equal); methodology (equal); project administration (equal); resources (equal); software (equal); supervision (equal); validation (equal); visualization (equal); writing – original draft (equal); writing – review and editing (equal). **Hua Zhang:** Conceptualization (equal); data curation (equal); formal analysis (equal); resources (equal); software (equal). **Shibiao Chen:** Conceptualization (equal); data curation (equal); formal analysis (equal); funding acquisition (equal); resources (equal); software (equal); supervision (equal).

## FUNDING INFORMATION

The project was funded by Department of Science and Technology of Jiangxi Province 20212BAG70034.

## CONFLICT OF INTEREST STATEMENT

The authors have nothing to disclose.

## Data Availability

Data available on request from the authors. The data that support the findings of this study are available from the corresponding author upon reasonable request.
